# Matching the green wave: growing season length determines embryonic diapause in roe deer

**DOI:** 10.1098/rspb.2024.2903

**Published:** 2025-05-21

**Authors:** Johanna Kauffert, Christian Ehrmantraut, Peter Mikula, Piotr Tryjanowski, Anette Menzel, Andreas König

**Affiliations:** ^1^ Professorship of Ecoclimatology, TUM School of Life Sciences, Freising, Bayern, Germany; ^2^ Wildlife Biology and Management Unit, TUM School of Life Sciences, Freising, Bayern, Germany; ^3^ Czech University of Life Sciences Prague Faculty of Environmental Sciences, Praha, Czech Republic; ^4^ Institute for Advanced Study, TUM, Garching, Bayern, Germany; ^5^ Department of Zoology, Poznań University of Life Sciences, Poznań, Poland

**Keywords:** embryonic diapause, length of season, phenology, phenotypic quality, proportional hazard model, roe deer

## Abstract

The roe deer (*Capreolus capreolus*) is Europe’s most widespread ungulate, notable for its unique trait of embryonic diapause (delayed blastocyst implantation after mating) and an ongoing debate regarding how climate change affects its parturition timing. Given the relatively constant timing of the rut, roe deer could cope with advancing greening by adjusting its diapause end. Here, we bridge the gap on factors influencing roe deer’s diapause by analysing 390 uteri from legally hunted roe deer females in Germany (2017–2020), which we macroscopically examined for the presence of visible embryonic tissue to retrospectively identify the diapause end date. By employing a marginal Cox proportional hazard model, we tested associations between female phenotypic attributes, environmental conditions and the probability of ending embryonic diapause prematurely. Our results confirmed that high-quality, well-conditioned and prime-aged females tend to terminate embryonic diapause earlier. We also demonstrated for the first time that on a population-averaged level, the growing season length in the year of conception significantly influences the diapause timing, even explaining the much-debated shifts in parturition dates in roe deer over the last seven decades. Increased knowledge of mechanisms involved in embryonic diapause may also help decipher embryo–maternal interactions in general, including *in vitro* fertilization.

## 1. Introduction

Changes in the timing of seasonal activities of plants and animals constitute significant ecological impacts of climate change [1–5], possibly leading to trophic mismatches [6,7]. In seasonal environments, animals time high energy-demanding events in their reproductive cycle, such as parturition and rearing, to the seasonal peak of abundance in high-quality food [8–10], using food availability, temperature and photoperiod as cues [11–13]. However, knowledge about adaptive responses is surprisingly elusive, especially for long-living mammals, such as ungulates [14,15]. Red deer, bighorn sheep and reindeer have shown trends towards earlier births in recent years, but the extent to which these changes result from reduced gestation length and earlier conception varies among species and is not yet fully understood [14–19].

**Figure 1.**
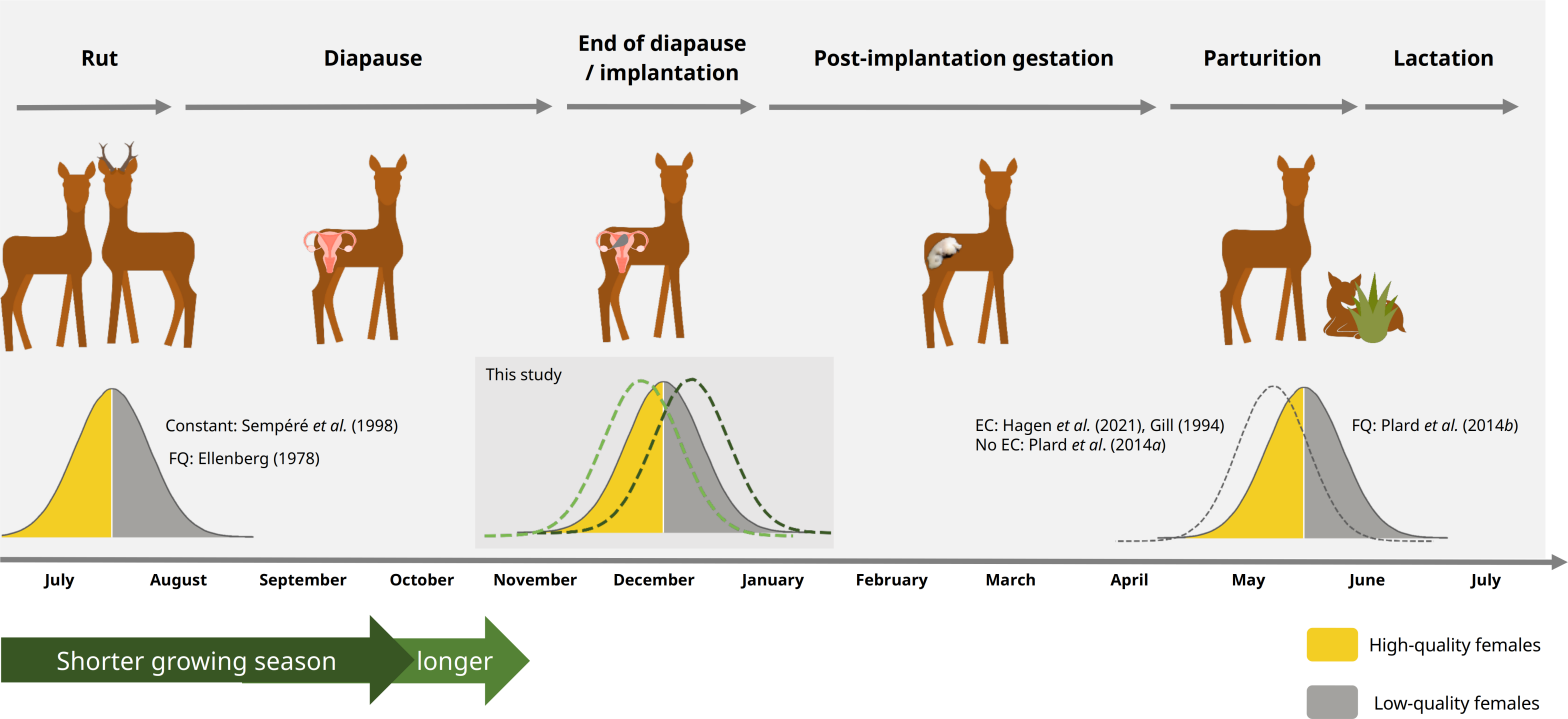
Reproductive cycle of roe deer with embryonic diapause. Female quality (FQ) influences the timing of rut and parturition; for example, high-quality females mate and give birth earlier. Whereas the timing of the rut is constant, there are contrasting findings on the altered timing of parturition and it is potentially driven by environmental conditions (EC) in the year of conception. Hypotheses depicted on the end of diapause tested in this study investigate, for the first time, the potential effects of environmental conditions in the year of conception and female quality and condition.

In contrast, the parturition timing of roe deer (*Capreolus capreolus*) is reported both to advance [20–22] or to be invariant to environmental changes [23,24]. These contradictory findings on phenotypic variations pose challenges since this species has a unique reproductive strategy among ungulates. It is the only representative of the artiodactyl clade known to undergo an (obligate) embryonic diapause [25]. After mating in summer, the blastocyst cell growth rate markedly reduces [26,27] and enters embryonic diapause, delaying its implantation for around 4–5 months [28–30]. At the end of embryonic diapause, cell growth accelerates during the elongation phase and implantation finally takes place [31] (figure 1). So far, the underlying physiological mechanisms have not been fully identified [30,32,33]. While there is some evidence of nutrient sensing [34], the end of embryonic diapause is believed to be influenced by photoperiod, aligning with the winter solstice in late December. However, the bandwidth of embryonic developmental stages during this period varies greatly among individuals [27,33,35,36].

As an income breeder [37], roe deer rely on the availability of highly digestible nutrition throughout the lactation period [38–40] and hence should, ideally, match parturition to the onset of spring. While earlier parturition timing in response to advancing greening was detected in Switzerland and southern Germany [20–22] and related to forsythia flowering dates of the year of conception [21], parturition timing was insensitive to environmental cues in eastern France [24]. Instead, in France, the time-invariant phenotypic quality of females mattered, specifically that high-quality females gave birth earlier than low-quality females [41]. Similarly, heavier female yearlings have been reported to mate earlier [42].

Giving birth early can enhance the likelihood of successful weaning and the offspring’s chances of survival [41,43], and it allows better synchronization with the advancing green wave. Thus, embryonic diapause could be the most crucial lever in adjusting roe deer’s reproductive effort [44]. Shedding light on mechanisms, timing and extent of variations in roe deer’s reproductive cycle may also help reduce fawn mortality during spring mowing [45]. Up to 8% of the fawns are estimated to be killed due to mowing grassland and fodder crops [46] despite rescue measures farmers and volunteers apply. Whereas an advance in plant flowering and greening with anthropogenic warming and altered mowing dates are well-established [47,48], there is a considerable knowledge gap on the timing of particular events in the reproductive cycle of roe deer. We hypothesize that on a population-averaged level, prime-aged rather than subadult or senescing females and high-quality and well-conditioned females end the embryonic diapause earlier. Additionally, we expect environmental factors in the year of conception to influence the timing of the end of embryonic diapause. We further expect that this potential effect aligns with the shifts in reported parturition phenology over the last 70 years in southern Germany.

This study used a dataset of 390 roe deer uteri of legally hunted females in Bavaria, Germany, between 2017 and 2020. The uteri were macroscopically examined for the presence of visible embryonic tissue to back-calculate the day of the end of embryonic diapause using the embryo’s crown-rump length (CRL [36]). We then used a marginal Cox proportional hazard model to relate the probability of ending embryonic diapause to female phenotypic quality, condition and age (compare [41]), the growing season’s length and warm and cold spells of temperature in November in the year of conception.

## 2. Material and methods

### (a) Study area

Roe deer females were harvested in various subunits of the Bavarian State Forest Enterprise (Bayerische Staatsforsten AöR) and private hunting units in Bavaria, in the southeastern part of Germany. Due to pronounced climatic and land-use gradients in Bavaria, we clustered the habitats into three biogeographical regions (Alps, Prealps and Scarplands), according to Ssymank [49]. Yearly mean temperatures differentiate between these regions from 6.72°C (s.d. = 1.76°C) for the alpine regions, 9.7°C (s.d. = 0.54°C) for the Prealps and 9.95°C (s.d. = 0.48°C) for the Scarplands [50].

### (b) Data collection and laboratory work

We examined the carcasses of 390 female roe deer legally hunted during the winters of 2017–2020. These deer were either hunted between 15 October and 15 January during the regular hunting season (*n* = 377) or hunted outside the regular hunting season until the end of February with special hunting permits (*n* = 13). Data were gathered along with details about the location and date of harvest. The total body weight, the eviscerated weight and the mandible length were measured shortly after their demise. The lower jaw’s M1 tooth was extracted to apply the cementum annuli method for age determination [51]. After field dressing, the uteri and ovaries were extracted, frozen at −18^◦^C and later examined for macroscopically visible embryonic tissue. If embryonic tissue was macroscopically detectable, the embryonic diapause had concluded at the time of harvest [29,52]. The CRL of any embryo was measured with calipers. We back-calculated the day of the end of embryonic diapause with the growth rates laid out in Bubenik [53]: CRL up to 25 mm: 1.8 mm growth per day, CRL of 25−50 mm: 2 mm growth per day, CRL of 50−100 mm: 2.5 mm growth per day and CRL over 100 mm: 2.9 mm growth per day.

Otherwise, without embryonic tissue, we considered the sample as right-censored data, as the diapause could have either ended after its demise, the female was not pregnant or had a spontaneous abortion. We only included females that were >1.5 years of age, had visible embryonic tissue or visible corpora lutea (endocrine glands involved in ovulation and early pregnancy [54]) and were harvested from 15 October until the end of February.

#### (i) Female phenotypic quality, condition and age

**Figure 2.**
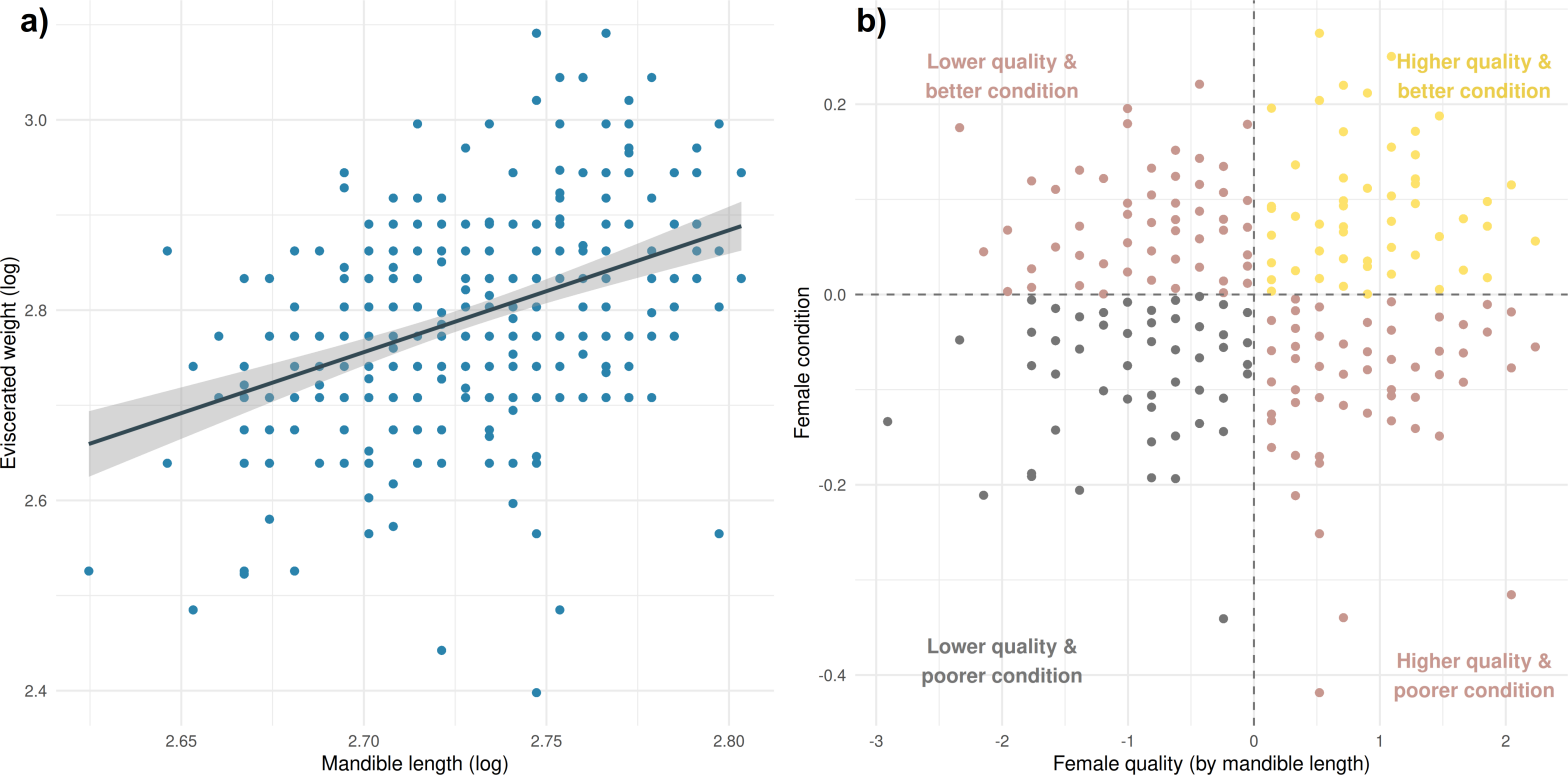
(a) Linear regression between mandible length (cm) and eviscerated weight (kg) (both log-transformed) (Adj. *R*^2^ = 0.162 given by the linear model; *n* = 330). (b) QCC of females into (1) better condition and higher quality (*n* = 81), (2) better condition and lower quality or poorer condition and higher quality (*n* = 174), and (3) poorer condition and lower quality based on the female’s quality (*n* = 75) given by the mandible length and the female’s condition given by the residuals from the regression model of (a).

To test the link between female phenotypic attributes and the probability of ending embryonic diapause early, we categorized roe deer females into three groups based on their quality and condition (from here: quality condition classification (QCC)). For the condition part, we calculated an index using the residuals of the linear regression between the body mass and the mandible length of each individual [44]. Analogous to Hewison & Gaillard [44], we used the log scale of the two variables: body mass and mandible length (figure 2a). The body mass was estimated by the eviscerated weight of the female to reduce any biases related to recent food intake. The mandible length is a proxy for early-life conditions, which we used as a rather time-invariant proxy for general female quality and performance in the quality part of our QCC [55].

Based on the female condition index (estimated as residuals from the model with body mass as the response variable and mandible length as predictor) and the female quality index (standardized mandible length), we grouped the females into three groups in our QCC (figure 2b): (1) better condition and higher quality (*n* = 81), (2) better condition and lower quality or poorer condition and higher quality (*n* = 174), and (3) poorer condition and lower quality (*n* = 75). We additionally added a three-level age class to our model to test if senescing (> 7 years; *n* = 26) and subadult (< 2 years; *n* = 91) females end embryonic diapause later than prime-age (≥ 2 to ≤ 7 years; *n* = 213) females [41,44]. The age of the females was calculated based on the cementum annuli method (cementum is the specialized connective tissue surrounding the root of each tooth, forming seasonal layers appearing as rings) on the lower M1 at the time of harvest. Due to some missing data on weight, mandible length or age, models including female QCC and age were calculated on a reduced sample (*n* = 330).

#### (ii) Environmental conditions

For testing the influence of environmental conditions in the year of conception on the timing of the end of embryonic diapause, we used the length of the growing season (LOS) and temperature spells in November, characterizing the vegetation growing conditions in the year of conception and favourable/unfavourable temperature conditions in the early implantation phase, respectively. The LOS (days) was defined by the period of the beginning of flowering of forsythia (*Forsythia suspensa* (*Thunb.*) Vahl.) to the autumn leaf fall of pedunculate oak (*Quercus robur* L.). Data were provided by the German Meteorological Service (DWD) as an interpolated raster (spatial resolution: 1000 m) [56]. The shortest recorded LOS for our study was 208 days, while the longest LOS lasted 240 days.

We retrieved daily mean temperature (°C) data from the E-OBS Gridded Dataset (v. 27.0) with a spatial resolution of 0.1° [57]. For the temperature spells, we compared the magnitude of deviation in mean temperature in November of the year of conception (standard deviations) to the respective baseline temperature means (1971−2000) of November [58]. By employing the magnitude of deviation from the mean, we likely yielded biologically more relevant results than using mean temperatures alone, as organisms tend to be more responsive to deviations from a mean state than to absolute values [58–61]. For both variables (LOS and November temperature spells), we calculated the median of the sampled raster values corresponding to each female’s respective location/hunting unit of harvest.

### (c) Statistical analyses

We used a marginal Cox proportional hazard model with the coxph function implemented in the survival v. 3.5−8 package in R v. 4.3.3 [62]. This semi-parametric model [63] is essentially a regression model commonly used in medical research to investigate the association between the survival time of patients and one or more predictor variables, such as potential treatments [64]. Like decreasing human survival probabilities with time, the likelihood of not ending the diapause for roe deer decreases during winter. The employment of this survival model enabled us to take full advantage of all observations (*n* = 390), including the right-censored ones for which the potential end of embryonic diapause was after harvest. This would be analogous to patients dropping out of the trial (no follow-ups) before experiencing the event of interest (e.g. relapse or death) but are still contributing with their respective survival times, which are ergo greater than the observation time, to the model [64,65].

To accommodate potential clustering within the biogeographical regions and to derive pooled effects, we estimated the standard error by utilizing the grouped jackknife variance estimate (robust variance) within the cluster term of *coxph* [66]. We decided not to use mixed-effect models as they frequently rely on unverifiable assumptions concerning the distribution of the random effects [67,68]. We tested for the proportional hazards assumption using the Schoenfeld residuals with *cox.zph* function. We report model test statistics for the likelihood ratio test (LRT), Wald test and score test. While the LRT and score tests assume independence of observations in clusters (subregions), the Wald test does not.

Due to the different sample sizes (*n* = 390, but for female phenotypic attributes and age *n* = 330), we first employed our variables independently using three models: we related the time of the end of embryonic diapause to female QCC and age (Model 1), to the LOS (Model 2) and to the temperature spells in November (Model 3). In Model 4, we combined all variables based on the smaller sample size associated with female phenotypic attributes and age. We checked for correlations between the predictor variables with the Kendall rank correlation coefficient due to non-normality of some predictor variables, revealing generally low to intermediate correlation between predictors (*r* < 0.5 in all cases) (electronic supplementary material, figure S1), justifying the use of all predictors in the same model. Based on Models 1 and 2, we additionally predicted the survival curves for the end of diapause for different QCCs and based on varying LOS for a representative area of our study areas (Kaisheim in Swabia/Scarplands).

## 3. Results

**Figure 3.**
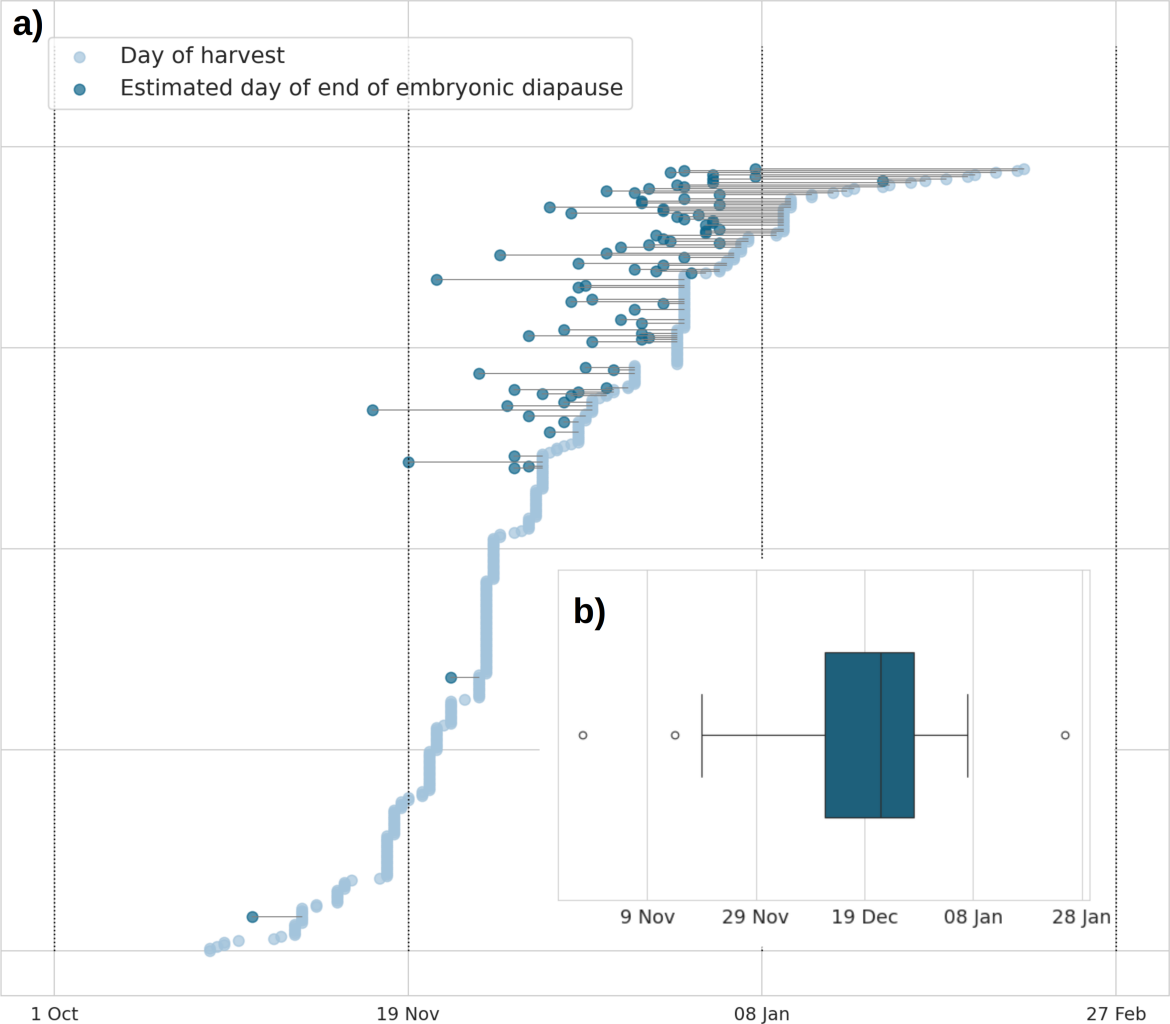
(a) Date of harvest/death of each female (*n* = 390) of the study and its estimated date of end of embryonic diapause (if embryonic diapause had ended before demise; *n* = 80). (b) Boxplot of the estimated date of end of embryonic diapause (if embryonic diapause had ended before demise; *n* = 80).

By the day of their demise (∼hunting date), 20.5% (*n* = 80/390) of the females had ended their embryonic diapause, while 79.5% (*n* = 310/390) of the females contributed as right-censored observations to the models as they were harvested before their potential end of embryonic diapause. The earliest day of the end of embryonic diapause was recorded as 28 October, and the latest date was 25 January. Eighty per cent of the females that had ended the embryonic diapause did so between 4 December and 1 January (figure 3).

**Figure 4.**
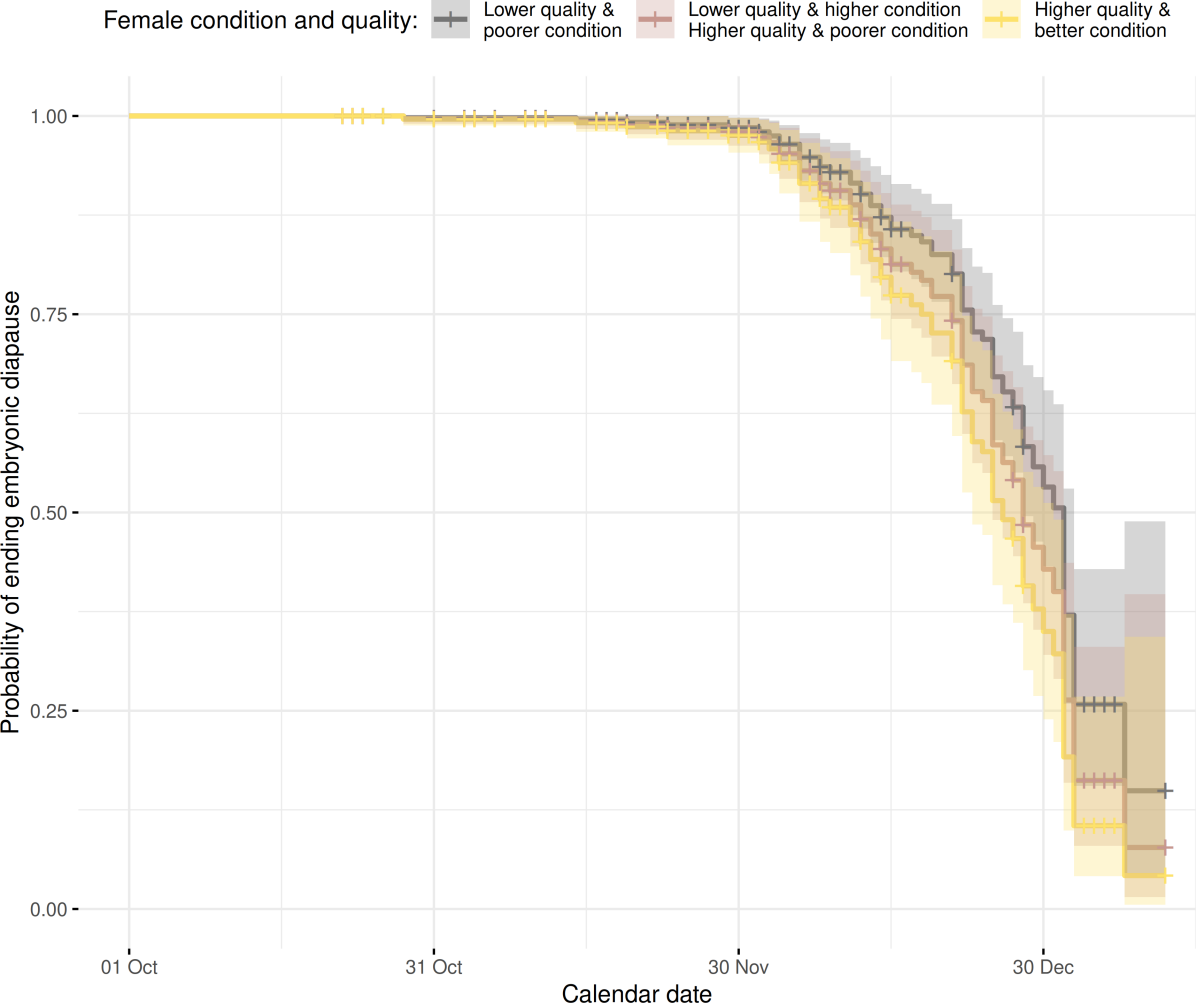
Model predictions (Cox proportional hazard model) for the probability of ending embryonic diapause in relation to the QCC (refer to Model 1, table 1; *n* = 330 with 67 events), while focusing only on prime-aged females (≥2 to ≤7 years).

The influence of female phenotypic attributes and age was assessed by classifying females according to their condition and quality (QCC) and age class. On a population-averaged level, high-quality females in good condition ended embryonic diapause earlier than their lower-quality and poorer-conditioned counterparts (Model 1: Hazard ratio (HR) = 1.664, *p*‐value ≤ 0.001; figure 4 and table 1). Similarly, females being either in better condition or of higher quality increased the chances of ending diapause earlier in comparison to low-quality and poorer-conditioned females (Model 1: HR = 1.343, *p*‐value ≤ 0.001; figure 4). The age of the females was also linked to the time of ending embryonic diapause. Senescing females ended diapause significantly later than prime-age females (Model 1: HR = 0.822, *p*‐value = 0.046). We had mixed results on the significance of Model 1 (a significant Wald test, whereas the LRT and score tests suggested that the explanatory variables were not significantly improving the model). Thus, there might be some uncertainty about whether the effect of our proxies for female phenotypic quality, condition and age is genuinely significant (table 1, Model 1).

**Table 1.** The effects of female quality and condition (QCC), age and environmental parameters on the probability of ending the embryonic diapause in roe deer. Each model represents results from a Cox proportional hazard model with a grouped jackknife variance estimate for the cluster of the subregion. Females are classified based on their quality and condition (QCC) and their age. LOS is the length of the growing season in the year of conception and November Spell is the standard deviation of mean November temperatures compared with a long-term average. The reference group for QCC is lower condition and poorer quality, and prime age for the age class. We report the regression coefficients (*β*) and their standard error (s.e.), hazard ratio (HR), robust standard errors (robust s.e.), *z*-value and *p*‐value. Asterisks indicate the level of significance: *<0.05, ***<0.001. Correlation analysis between the predictor variables is given in the electronic supplementary material.

model		*β*	HR	s.e. (*β*)	robust s.e.	*z*	*p*‐value	
1	better condition and poorer quality or poorer condition and better quality	0.295	1.343	0.327	0.071	4.18	<0.001	***
	better condition and better quality	0.509	1.664	0.369	0.118	4.3	<0.001	***
	age class: senescing	−0.196	0.822	0.448	0.098	−2	0.046	*
	age class: subadult	−0.181	0.835	0.301	0.108	−1.67	0.094	.
	*n* = 330, events = 67; LRT = 2.64, *p* = 0.6;	Wald test = 28.3, *p* ≤ 0.001;			score (log rank) test = 2.61, *p* = 0.6			
2	LOS	0.045	1.046	0.016	0.001	56.5	<0.001	***
	*n* = 390, events = 80; LRT = 8.87, *p* = 0.003;	Wald test = 3196, *p* ≤ 0.001;			score (log rank) test = 8.58, *p* = 0.003			
3	sigma of November Spell	0.187	1.206	0.397	0.373	0.5	0.62	
	*n* = 390, events = 80; LRT = 0.22, *p* = 0.6;	Wald test = 0.25, *p* = 0.6;			score (log rank) test = 0.22, *p* = 0.6			
4	better condition and poorer quality or poorer condition and better quality	0.414	1.512	0.334	0.081	5.13	<0.001	***
	better condition and better quality	0.517	1.675	0.38	0.051	10.19	<0.001	***
	age class: scenescing	−0.365	0.694	0.46	0.166	−2.21	0.027	*
	age class: subadult	−0.148	0.862	0.301	0.124	−1.19	0.234	
	LOS	0.032	1.033	0.019	0.002	18.2	<0.001	***
	sigma of November Spell	0.291	1.337	0.546	0.125	2.32	0.02	*
	*n* = 330, events = 67; LRT = 6.19, *p* = 0.4;	Wald test = 262, *p* ≤ 0.001;			score (log rank) test = 6.22, *p* = 0.4			

On the population-averaged level, the LOS in the year of conception positively influenced the ending of embryonic diapause (Model 2: HR = 1.046, *p*‐value ≤ 0.001, table 1). In support of all test statistics (LRT, Wald test and score test), a greater LOS increased the chances of females ending diapause earlier. In contrast, we did not find any significant support for an influence of the November temperature spells (Model 3: HR = 1.206, *p*‐value = 0.62, table 1). In Model 4, all predictor variables (for their mutual correlation, see electronic supplementary material, figure S1) are incorporated into a single model (table 1). Interestingly, the effect of November temperatures was more pronounced in the model incorporating female quality, LOS and November temperature spells (Model 4: HR = 1.337, *p*‐value = 0.02, table 1), hinting at the possible influence of comparably high November temperatures on an earlier end of embryonic diapause.

**Figure 5.**
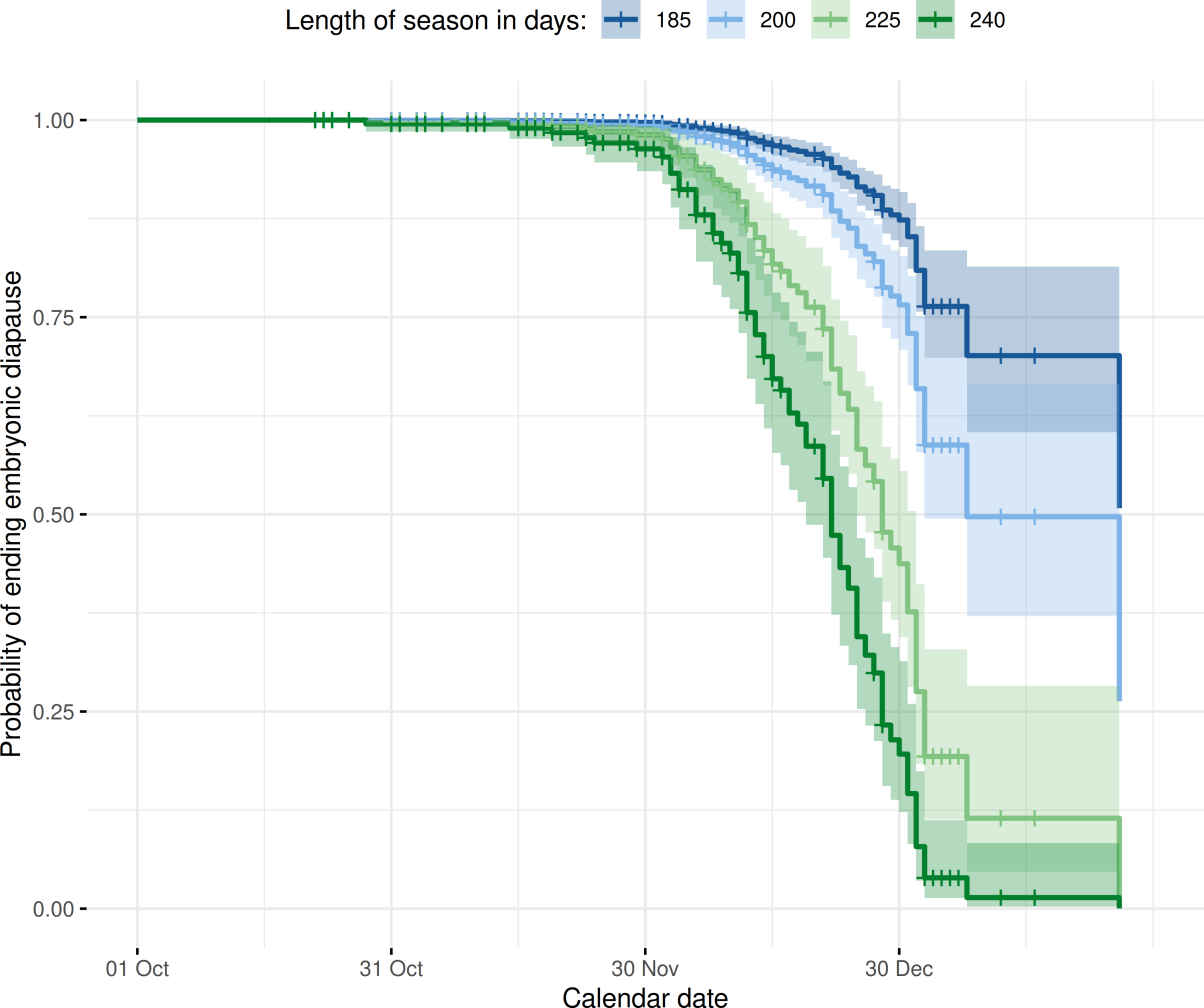
Model predictions (Cox proportional hazard model) for the probability of ending embryonic diapause in relation to the varying LOS in the year of conception (refer to Model 2 table 1; *n* = 390 with 80 events). We used four different LOS scenarios, ranging from 185 to 240 days, thus mirroring conditions in the 1950s to recent years.

When predicting the response in the timing of the end of embryonic diapause for different LOS to assess the effect sizes (figure 5), it turned out that with greater LOS, the date when females ended their embryonic diapause could occur remarkably earlier. Specifically, with a shorter LOS (e.g. 185 days, which is equivalent to a mean LOS in 1951−1960 for the study region), the 75% threshold (survival probability to which date females have their embryonic diapause) is reached by 6 January. In contrast, when the LOS is longer (e.g. 200 days, ∼ mean LOS between 1991 and 2000), the 75% mark was reached as early as 30 December, while with a LOS of 225 days (∼ mean LOS between 2011 and 2020), it occurs another 10 days earlier (20 December). With a LOS comparable to recent years (240 days), the 75% threshold could already be reached on 12 December. Hence, for an observed lengthening of the growing season from the 1950s to recent years, the corresponding model predicted 26 days advance in the 75% likelihood threshold for ending the diapause in Bavaria. We compared this number with the shifts of reported roe deer’s reproductive cycle timings, specifically with the earlier start of parturition timing and finding dates of fawns in southern Germany. In historical periods (1938−1945), ∼60% of fawns were found in May, and the median day of parturition for southwestern Germany was ∼1 June [69]. In recent years (2020−2022), approximately 76% of fawns were found in May and the median birth date was 15 May [22,45] (also compare [21]). This would entail a shift in parturition timing of 18 days between 1938−1945 and 2020−2022, which largely corresponds to our predicted shifts in termination of the diapause.

## 4. Discussion

To the best of our knowledge, we found evidence that embryonic diapause in roe deer is tied to environmental drivers. Specifically, we found on a population-averaged level a link between the timing of the end of embryonic diapause and the LOS in the year of conception. Under the assumption of a relatively fixed post-implantation gestation length [41], roe deer females should shift parturition timing in response to climate change and the consequential earlier onset of spring [20–22]. Our findings on the diapause ending support Hagen *et al.* [21] results on parturition timing, where the timing of flowering of forsythia in the year of conception could explain up to 37% of the variance of mean parturition date. Furthermore, the predicted shifts in the timing of the end embryonic diapause due to an extended growing season largely correspond with the changes in parturition timing observed between 1938−1945 and 2020−2022 in southern Germany [21,22,45,69]. In contrast, we did not find a significant relationship between November temperature spells and the end of embryonic diapause, although our associations also pointed in the direction that poor weather conditions during the implantation phase were suggested to be detrimental concerning roe deer’s fecundity (indirectly or at an inter-population level [44,70]).

Roe deer’s widespread distribution in Europe, its adaptability to new habitats, such as agricultural landscapes [71,72], and its presence in Europe for at least 600 000 years highlight its general adaptive capacity [73,74]. Shifts in the termination of embryonic diapause may be another adaptation enabling roe deer to cope with changing environmental conditions. The insights from relatively well-studied parturition timing indicate that this trait varies markedly between regions in Europe (mean parturition date ranges from April in Spain to mid-June in Sweden) [75]. Observations on parturition timing also revealed no simple latitudinal gradient but adjustments to local conditions [69,75,76], even resulting in shifts at a regional scale [42,76]. Also, within the same population, shifts in parturition timing were observed in response to changes in habitat quality, entailing earlier births with periods of good habitat quality [77].

Alternatively, instead of adjusting the start of the post-implantation gestation (i.e. ending of diapause) as outlined before, one could argue that the observed variation in parturition timing results from high variability and temporal shifts in mating. Yet, while the gestation and parturition periods have been found to change considerably in time and space and also to vary between individuals in one population [75], little temporal variability has been observed in rutting times [25,42,78]. Sempéré *et al.* [79] suggested that the female oestrus is induced by decreasing day lengths in late July, mirrored by seemingly synchronized rutting times across Europe [80] with an indication for slightly later rut at northern latitudes [81]. Furthermore, records from the eighteenth century (during the Little Ice Age) reported roe deer’s mating timing around St. Jacobi day (25 July) [82], which is within the earlier tail of rutting times observed in Bavaria nowadays (start: 22−23 July; peak: 3−4 August; end: 16−17 August [83]).

Phenotypic female quality in ungulates is an influential factor in the timing of reproduction [41]. Our results suggest that phenotypic quality and condition are also linked to the timing of the end of embryonic diapause in roe deer. In contrast, phenotypic quality or condition was not shown to affect implantation failure rates [44], but female senescence was shown to increase those rates [44,84]. Likewise, we found that senescing females end embryonic diapause later than prime-age females. However, due to our reduced sample size on female quality, condition and age and the (partially) non-significant test statistics, further data are needed to explore this link in more detail. Still, our findings partially align with Plard *et al.* [41], who similarly found that high-quality females show a proclivity to give birth earlier than low-quality females. In previous studies, rutting times were also related to body weight and age (heavier yearlings and older females mate or ovulate earlier, respectively [42,85]; see summarizing scheme in figure 1 bottom). Interestingly, most females (80%) in our sample ended embryonic diapause within 28 days, which is analogous to the estimated length of the rutting period in Bavaria [83]. Consequently, it seems plausible that females’ phenotypic attributes are decisive in the variation of the timing of reproductive events during the rut and consecutively during diapause and parturition on population levels [41].

Phenotypic attributes of females were not only found to be important in the reproductive phenology [41] and longevity [86] but also in its interplay with fecundity, pregnancy rates, reproductive success and litter size [44,84,87–90]. High-quality females were more likely to reproduce younger [88,91], rut/ovulate [42,85] and give birth earlier [41], increasing the survival chances of their offspring [92], have a potentially larger litter size [36,44] and live in non-hunted areas longer which, in turn, also influences the number of breeding attempts a female can undergo [86]. Thus, our finding of high-quality and well-conditioned females ending the embryonic diapause earlier aligns well with these other results and may even explain why they give birth earlier due to its glimpse into the former ‘black box’ of diapause.

From a wider taxonomic perspective, at least 130 species are known to undergo delayed embryo implantation, and it is hypothesized to be a reversible trait to adapt to unfavourable conditions during pregnancy and may even be an ancestral trait common to all mammals [93]. Delayed implantation is under lactational (e.g. house mouse *Mus musculus*) [94] or seasonal, mainly photoperiod (e.g. American mink *Neovison vison*) [95] control or even both (e.g. tammar wallaby *Macropus eugenii*) [96]. To what degree these species can also adjust their reproduction to environmental change has yet to be unravelled. In pinnipeds, changes in the reproductive phenology have been proposed to be linked directly or indirectly to climate change, such as in common seals (*Phoca vitulina*) [97] or grey seals (*Halicheorus grypus*) [98]. Yet, phenological shifts in grey seals have also been attributed to organismal mechanisms, such as changes in the age structure at the study site of this migrating species [99].

Roe deer’s constraints as an income breeder, relying on environmental conditions of the year prior to giving birth to potentially anticipate the conditions of the following spring, could entail detrimental consequences. Moreover, in animals mainly relying on plant phenological autumn cues (like Pyrenean chamois *Rupicapra pyrenaica pyrenaica*) [100], an increased mismatch could be expected since the rate of global change impacts on leaf phenology considerably differs between autumn and spring [3,48,101].

Our findings of linking the timing of the end of embryonic diapause for the first time to the LOS in the year of conception could help to understand the possible shifts of roe deer in relation to environmental changes and, ultimately, trophic mismatches. Since the phenotypic quality of females also plays a vital role in the reproductive cycle of roe deer, future studies concerning roe deer’s phenological shifts should pay special attention to possible interactions between environmental and organismal mechanisms to disentangle opposing or synergistic effects [98,102]. We hypothesize that, to a certain degree, the effects of female quality and condition could be a result of high-quality and/or well-conditioned individuals also mating earlier, but that individuals also experience a level of plasticity during their implantation phase which is mediated by environmental conditions (figure 1). In fact, a recent study proposed nutrient sensing as a potential key factor in regulating embryonic developmental pace, demonstrated by an increase in mTORC1-activating amino acids [34]. However, we could not directly test this because we analysed our data at a population level and could not derive individual-level responses [103]. Concerning the prevention of mowing death in spring by anticipating when to expect most of the young fawns in the meadows, careful consideration should be paid to the interactions between demographic parameters of the population, including selection by hunters and cohort effects [104,105], while monitoring environmental conditions in the year of conception leading to possible shifts in parturition timing. Yet, due to their plastic response to vegetation, roe deer in our study region are currently not suffering from a pronounced energy shortfall [39,40] and are adapting well to respective nutrient availabilities [106], making significant short-term temporal shifts in breeding phenology unnecessary.

Deciphering embryo–maternal interactions holds the promise to explain pregnancy losses and fertility and serve as a stepping stone towards artificial reproductive technology, i.e. for biodiversity preservation of nearly extinct animals [107]. Moreover, unravelling the black box of embryonic diapause could resonate beyond and even play a pioneering part in the research of diapause-like reversible drug-tolerant persister state of cancer cells [108,109]. Therefore, findings on mechanisms mandating delayed embryo development in model animal species that regularly exhibit embryonic diapause may also be useful in human and veterinary medicine.

## Data Availability

Data and code are permanently archived on Dryad [110]. Exact harvest locations cannot be published due to reasons of privacy rights of hunters and landowners. Supplementary material is available online [111].
